# Detectable HIV Viral Load in Kenya: Data from a Population-Based Survey

**DOI:** 10.1371/journal.pone.0154318

**Published:** 2016-05-18

**Authors:** Peter Cherutich, Andrea A. Kim, Timothy A. Kellogg, Kenneth Sherr, Anthony Waruru, Kevin M. De Cock, George W. Rutherford

**Affiliations:** 1 National AIDS/STI Control Programme, Ministry of Health, Nairobi, Kenya; 2 US Centers for Disease Control and Prevention, Division of Global HIV and Tuberculosis, Nairobi, Kenya; 3 University of California San Francisco, San Francisco, United States of America; 4 University of Washington, Seattle, United States of America; University of Athens, Medical School, GREECE

## Abstract

**Introduction:**

At the individual level, there is clear evidence that Human Immunodeficiency Virus (HIV) transmission can be substantially reduced by lowering viral load. However there are few data describing population-level HIV viremia especially in high-burden settings with substantial under-diagnosis of HIV infection. The 2^nd^ Kenya AIDS Indicator Survey (KAIS 2012) provided a unique opportunity to evaluate the impact of antiretroviral therapy (ART) coverage on viremia and to examine the risks for failure to suppress viral replication. We report population-level HIV viral load suppression using data from KAIS 2012.

**Methods:**

Between October 2012 to February 2013, KAIS 2012 surveyed household members, administered questionnaires and drew serum samples to test for HIV and, for those found to be infected with HIV, plasma viral load (PVL) was measured. Our principal outcome was unsuppressed HIV viremia, defined as a PVL ≥ 550 copies/mL. The exposure variables included current treatment with ART, prior history of an HIV diagnosis, and engagement in HIV care. All point estimates were adjusted to account for the KAIS 2012 cluster sampling design and survey non-response.

**Results:**

Overall, 61·2% (95% CI: 56·4–66·1) of HIV-infected Kenyans aged 15–64 years had not achieved virological suppression. The base_10_ median (interquartile range [IQR]) and mean (95% CI) VL was 4,633 copies/mL (0–51,596) and 81,750 copies/mL (59,366–104,134), respectively. Among 266 persons taking ART, 26.1% (95% CI: 20.0–32.1) had detectable viremia. Non-ART use, younger age, and lack of awareness of HIV status were independently associated with significantly higher odds of detectable viral load. In multivariate analysis for the sub-sample of patients on ART, detectable viremia was independently associated with younger age and sub-optimal adherence to ART.

**Discussion:**

This report adds to the limited data of nationally-representative surveys to report population- level virological suppression. We established heterogeneity across the ten administrative and HIV programmatic regions on levels of detectable viral load. Timely initiation of ART and retention in care are crucial for the elimination of transmission of HIV through sex, needle and syringe use or from mother to child. Further refinement of geospatial mapping of populations with highest risk of transmission is necessary.

## Introduction

The relationship between viremia and Human Immunodeficiency Virus (HIV) transmission at the individual level is well established [1–3]. Furthermore, observational and clinical trial data indicate that viral load (VL) suppression using antiretroviral therapy (ART) significantly reduces HIV transmission through sex and breastfeeding [3, 4]. Consequently, population-level viral load suppression is now a global public health imperative.

Metrics to describe aggregate viral load include community viral load (CVL) and population viral load (PVL). These metrics could be described either as means (mean VL) or medians (median VL). CVL as a summary measure of those HIV-infected persons already engaged in HIV care is limited by the lack of comparability of populations that may have the same level of CVL but whose underlying HIV transmission risk is modified by disparate HIV prevalence. Additionally, the VL of those unaware of their HIV infection needs to be taken into account for CVL to measure risk of population-level HIV transmission accurately [5]. However, PVL, the proportion of HIV-infected persons with detectable or, conversely, undetectable VL is increasingly considered as an appropriate measure for treatment and prevention programs and for evaluating ART outcomes at the population level, as it addresses the limitations of CVL [6, 7].

In sub-Saharan Africa VL measurements are largely limited to diagnosing and monitoring treatment failure, and their use for surveillance of population-level infectivity is uncommon. Furthermore, previous studies have been limited to people on ART as well as national HIV and lab surveillance registries [8–12].

Kenya has a generalized HIV epidemic and a high burden of disease. In 2012, an estimated 1·4 million adults were living with HIV/AIDS, of whom an estimated 90,000 were newly infected that year [13]. Kenya is among high HIV-burden countries that have made commitments to achieve 90% viral suppression among people on ART and tracking the implementation of this policy goal is vital. The 2^nd^ Kenya AIDS Indicator Survey (KAIS 2012) provided a unique opportunity to evaluate the impact of ART coverage on viremia and to examine the risks for failure to suppress viral replication. This study examined population-level HIV viral load suppression using data from this nationally representative survey.

## Methods

### Study design

KAIS 2012 was a nationally representative, cross-sectional survey of household residents in Kenya. The details of the study have been described elsewhere [14]. Briefly, KAIS 2012 surveyed household members, administered questionnaires, and drew serum samples to test for HIV. For those found to be HIV-infected, PVL was measured.

Eligible individuals were enrolled between October 2012 and February 2013. This paper examines a sub-sample of Kenyans aged 15–64 years who had laboratory-confirmed HIV infection.

### Data collection

The interview captured demographic data, prior HIV testing and ART history. Co-morbidity with tuberculosis (TB) was measured by self-reported lifetime history of a TB diagnosis by a doctor or health professional. Participants who reported having been sexually active in the year before the interview were asked for the number of sex partners in the past 12 months. In addition, the survey asked for partner-specific sexual behaviors for all reported sex partners in the past 12 months, including condom use. Consistent condom use was defined as if the participant reported “a condom used all the time” with every partner during sexual intercourse. Sexually active respondents were also asked if they had ever exchanged money, gifts, or favors (either received or given) for sex. Discordant partner status was obtained for partners who resided in the same household and who consented to both the KAIS survey and HIV serologic testing.

We also captured the geospatial location of households to enable us determine the distribution of HIV infection and HIV viremia across counties and ten programmatic regions in Kenya.

### Laboratory measurements

We screened blood specimens for HIV antibody using the Vironostika HIV-1/2 UNIF II Plus O Enzyme Linked Immunoassay (bioMérieux, Marcy l’Etoile, France), a fourth-generation assay. Non-reactive specimens were reported as HIV-negative. Reactive specimens were confirmed with the Murex HIV·1·2·O HIV Enzyme Immunoassay (DiaSorin, SpA, Saluggia, Italy), also a fourth generation assay. Those that were reactive on both assays were reported as having final HIV-positive results. Samples with discordant HIV results were re-tested using the same algorithm and, if discordance persisted, the samples were tested for the presence of HIV antigen using the Roche v1·5 polymerase chain reaction (COBASOBAS AMPLICOR HIV-1 Monitor Test, version 1·5, Roche Molecular Diagnostics, Pleasanton, California, United States (US)). We tested all final HIV-positive specimens for HIV-1 RNA concentration using the Abbott M2000 Real-Time HIV-1 Assay (Abbott Laboratories, Abbott Park, Illinois, US). Each HIV-positive specimen contributed one VL count. Specimens with PVL <550 copies/mL, the minimum concentration detectable on the assay, were classified as virologically suppressed. Quality control for PVL was conducted on ten percent of samples of HIV-infected patients at the Centers for Disease Control and Prevention (CDC) laboratory in Kisumu, Kenya. HIV-positive specimens were tested for the presence of antiretroviral therapy (ART) using High Performance Liquid Chromatography coupled to Tandem Mass Spectrometry. CD4+ T-cell count measurements were done using the BD FACSCalibur^™^ flow cytometer (Becton Dickinson BioSciences, San Jose, California, US). However, 1254 specimens hemolysed during transit from the field to the laboratory and1135 participants provided a dried blood spot only from which we could not measure CD4+ T-cell counts. Therefore, 54·0% of HIV-reactive specimens could not be subjected to CD4+ T-cell count testing at the National HIV Reference Laboratory.

### Data analysis

In this paper, we examine the predictors of HIV viremia in Kenyans aged 15–64 years. Our principal outcome variable was unsuppressed HIV viremia, defined as a PVL ≥550 copies/mL. Self-reported awareness of HIV status, current HIV care attendance, and current ART use was augmented to include results of ART presence in the samples. Therefore, as an example, if a respondent had ART detected in the blood, they were classified as “currently taking ART” regardless whether they self-reported ART use or not. Furthermore, we assumed that if ART was detected, participants were aware of their HIV infection and were in HIV care. All estimates were adjusted to account for the KAIS 2012 cluster-sampling design and survey non-response using the SURVEYFREQ procedure in SAS version 9·3 (SAS Institute Inc., Cary, North Carolina, US). Appropriate domains were used for all analyses. In bivariate analysis, population proportions and 95% confidence intervals (CI) were reported for categorical variables. We examined the overall prevalence of unsuppressed HIV viremia at PVL ≥550 copies/mL. We also investigated the association between unsuppressed HIV viremia and selected socio-demographic, behavioral, and clinical characteristics for those reporting current ART use and those not on ART. Variables found to be significant at a p-value <0·1 in bivariate analysis were entered in a multivariable logistic regression model using the SURVEYLOGISTIC procedure in SAS to determine characteristics that were independently associated with HIV viremia. Variables were removed using a backward elimination process. Variables retained in the final model at a p-value of <·05 were considered significant predicators and reported.

The weighted mean and median VL estimates with corresponding 95% CI and interquartile range (IQR) was calculated using the SURVEYMEAN procedure in SAS. The value used for infected persons with a VL below the lowest detectable limit of <550 copies/mL was zero.

### Geospatial analysis

We also determined the distribution of PVL across ten administrative and HIV programmatic regions in Kenya and calculated the ratio of those who were virally suppressed compared to those who were not. The ratio was calculated by dividing the weighted proportion of viremic adults by the proportion of adults who were virally suppressed. We interpreted a value of >1 as an indication that the population size of viremic adults was greater than adults virally suppressed. Conversely, we interpreted a value <1 as indicating that the population size of viremic adults was smaller than those who are virally suppressed.

### Ethical considerations

We obtained verbal informed consent from the study participants. Participants aged 15–17 provided verbal assent after obtaining verbal consent from their parents or guardians. Verbal consent is a standard practice for household surveys in Kenya and was preferred to ensure completeness and quality of the informed consent process. The study interviewers signed the consent forms as an attestation of informed participant consent. The Kenya Medical Research Institute Ethical Review Committee, the US CDC Institutional Review Board, and the Committee on Human Research of the University of California, San Francisco reviewed and approved the KAIS 2012 protocol and the consent procedures.

## Results

### Predictors of detectable viremia

Of the 648 HIV-infected persons, VL data were available for 617 (95·8%). Overall, the percent of infected Kenyans aged 15–64 years with detectable VL was 61·2% (95% CI: 56·4–66·1) (Table 1). The base_10_ median (IQR) and mean (95% CI) VL was 4,633 (0–51,596) and 81,750 (59,366–104,134) copies/mL, respectively. There were no significant differences found in the percentage of persons with detectable viremia by sex, marital status, widowhood, educational level, residence, region, and wealth index. However, persons aged 15–29 years had a significantly higher percentage of detectable viremia at 79·4% (95% CI: 72·4–86·5) than persons aged 30–64 years at 53·9% (95% CI: 48·4–59·5) (p<0·0001). In addition, persons who were unaware of their infection had significantly higher levels of detectable VL (91·2% [95% CI: 87·4–94·9]) compared to those who knew of their infection (39·5% [95% CI: 33·5–45·5]) (p<0·0001). Also, those who reported they were diagnosed with TB were significantly less likely to have detectable VL (29·8% [95% CI: 20·0–40·1]) compared with those without a TB diagnosis (65·5% [95% CI: 60·3–70·6]). Those that were enrolled in HIV care were significantly less likely to have detectable virus compared to those not in care (35·1% [95% CI: 28·9–41·3] versus 90·4% [95% CI: 86·5–94·3]) (p<0·0001). Among 266 persons currently taking ART, 26·1% (95% CI: 20·0–32·1) had detectable viremia compared to 88·8% (95% CI: 85·0–92·6) (p<0.0001) among 351 persons not taking ART. The percentage of persons with detectable viremia did not differ by recent sexually activity or by the number of sex partners in the past 12 months. However, among sexually-active HIV-infected persons, the percentage with detectable virus was lower for those who used a condom at the most recent sexual intercourse and who consistently used condoms with all sexual partners (p<0·0001) (Table 1).

**Table 1:** Characteristics of HIV-infected persons and estimates of detectable viral load, Kenya AIDS Indicator Survey, 2012.

Variable	Numberdetectable VL^1^/Total	Weighted % (95% CI^2^)	p-value
**Overall**			
Detectable viral load^1^	382/617	61·2 (56·4–66·1)	
**Sex**			0·308
Men	121/183	64·3 (55·7–72·8)	
Women	261/434	59·4 (54·2–64·5)	
**Age-group (years)**			**<0·0001**
15–29	139/175	**79·4 (72·4–86·5)**	
30–64	243/442	**53·9 (48·4–59·5)**	
**Marital status**			0·932
Unmarried/not cohabitating	153/248	61·0 (53·8–68·1)	
Married/cohabitating	229/369	61·4 (55·5–67·2)	
**Ever widowed**			0·136
No	290/454	63·1 (57·3–68·9)	
Yes	92/163	55·7 (47·5–63·8)	
**Educational level**			0·120
No or incomplete primary	49/84	59·3 (47·3–71·4)	
Completed primary	148/212	67·5 (59·2–75·7)	
Secondary +	185/321	57·7 (51·4–63·9)	
**Residence**			0·391
Rural	225/353	63·0 (56·1–70·0)	
Urban	157/264	58·9 (52·3–65·4)	
**NASCOP region**			0·158
Nairobi	37/63	56·2 (42·2–70·1)	
Central	26/57	50·0 (34·7–65·3)	
Coast	43/63	68·6 (53·4–83·8)	
Eastern	35/73	45·2 (29·7–60·8)	
Nyanza	151/227	66·0 (59·4–72·7)	
Rift Valley	52/77	63·1 (47·3–79·0)	
Western	38/57	66·0 (52·0–80·0)	
**Wealth index**			0·349
Lowest	65/96	66·2 (53·2–79·2)	
Second	90/146	60·1 (49·1–71·0)	
Middle	80/129	62·5 (53·5–71·4)	
Fourth	97/151	65·7 (56·4–74·9)	
Highest	50/95	50·6 (37·7–63·6)	
**Aware of HIV infection**^3^			**<0·0001**
No	238/263	**91·2 (87·4–94·9)**	
Yes	144/354	**39·5 (33·5–45·5)**	
**TB co-infection**			**<0·0001**
No	357/544	**65·5 (60·3–70·6)**	
Yes	25/73	**29·8 (20·0–40·1)**	
Currently in HIV care^4^			**<0·0001**
No	263/293	**90·4 (86·5–94·3)**	
Yes	119/324	**35·1 (28·9–41·3)**	
**Currently taking ART**^5^			**<0·0001**
No	309/351	**88·8 (85·0–92·6)**	
Yes	73/266	**26·1 (20·0–32·1)**	
**Time in care**			**<0·0001**
Not in care	263/293	**90·4 (86·5–94·3)**	
<12 months	22/42	**48·6 (33·9–63·3)**	
12–23 months	20/37	**53·3 (34·8–71·9)**	
24+ months	56/174	**31·5 (23·0–40·0)**	
Unknown	21/71	**27·9 (17·0–38·9)**	
**Sexually active in past 12 months**			0·208
No	97/168	56·4 (47·4–65·4)	
Yes	285/449	62·8 (57·3–68·3)	
**HIV status of sex partner**			0·257
HIV-infected	77/117	59·8 (48·1–71·5)	
HIV-uninfected	47/85	57·0 (46·5–67·4)	
Partner not tested	161/247	66·6 (59·9–73·3)	
Not sexually active	97/168	56·4 (47·4–65·4)	
**Number of sex partners in past 12months**			0·444
No partners	97/168	56·4 (47·4–65·4)	
One partner	249/387	63·0 (57·0–69·1)	
2+ partners	36/62	61·8 (48·9–74·6)	
**Condom use at last sex**^6^ **(N = 449)**			**<0·0001**
Yes	83/170	**47·1 (39·6–54·7)**	
No	202/279	**73·1 (66·6–79·6)**	
**Consistent condom use with all partners in past 12 months**^6^**(N = 449)**			**<0·0001**
Yes	57/122	**44·8 (35·8–53·8)**	
No	228/327	**69·8 (63·8–75·9)**	
**Ever exchanged sex for money/gifts (N = 449)**			0·220
No	252/387	64·0 (58·1–69·9)	
Yes	33/62	56·0 (43·8–6·.3)	

NASCOP, National AIDS Control Programme. These regions correspond to former provinces of Kenya before the 2013 decentralization into counties.

^1.^ Detectable viral load defined as HIV RNA concentration ≥550 copies/mL.

^2.^ Confidence Interval.

^3.^ Self-reported HIV-infected with presence of ART in blood.

^4.^ Includes those with detectable ART in blood.

^5.^ Either by self-report or by presence of ART in blood.

^6.^ Among sexually active in past 12 months.

In bivariate analysis, younger age-group, lack of awareness of infection, no TB co-infection, not currently in HIV care, no current ART use, time in HIV care, lack of condom use at last sexual intercourse, and inconsistent condom use were significant predictors of detectable viral load at a p-value <0·1 and were included in multivariable analysis. In multivariate analysis, non-ART use, younger age, and lack of awareness of HIV infection were associated with significantly higher odds of detectable VL (data not shown). Persons who were not currently taking ART had almost 12 times as high adjusted odds ratio (aOR) of having detectable VL as those who were currently taking ART (aOR 11·8, [95% CI:6·3–22·2]). Compared to persons aged 30–64 years, infected persons aged 15–29 years had more than three times the adjusted odds (aOR 3·3, [95% CI:2·1–5·3]), and persons unaware of their infection had more than twice the adjusted odds of having detectable VL (aOR 2·2, [95% CI: 1·1–4·4]).

Among 266 persons who were currently taking ART, we observed significant differences in the proportion of detectable viremia across age groups: 22·3% among persons aged 30–64 years compared with 46·5% among persons aged 15–29 years (p<0·0001). However, a higher proportion of persons who reported missing an antiretroviral dose in the past 30 days (46·1%) had detectable virus compared to persons who had not missed a dose during the same time frame (23·4%) (Table 2). We did not find any differences in the proportion of persons with detectable viremia by time in HIV care or self-reported TB co-infection. Men and women who were taking ART had similar proportions of detectable viremia at 24·1% vs. 27·2%, respectively (p = 0·630) (Table 2).

**Table 2:** Predictors of detectable viral load^1^ among adults who are currently taking ART, Kenya AIDS Indicator Survey, 2012.

Variable	Number/ Total	Weighted % (95% CI^2^)	OR^3^ (95% CI^2^)	p-value	Adjusted OR^3^^,^^4^ (95% CI)	p-value
**Overall**	73/266	26·1 (20·0–32·1)	—			
**Sex**				0·630		
Female	53/194	27·2 (19·7–34·7)	1·2 (0·6–2·3)			
Male	20/72	24·1 (14·0–34·2)	Ref			
**Age category (Years)**				**0·005**		
15–29	20/43	46·5 (29·4–63·6)	**3·0 (1·4–6·6)**		**3·1 (1·4–6·9)**	**0·006**
30–64	53/223	22·3 (29·4–63·6)	Ref		Ref	
**Residence**				0·458		
Rural	42/145	28·2 (19·6–36·7)	1·3 (0·7–2·4)			
Urban	31/121	23·6 (15·0–32·1)	Ref			
**Marital status**				0·377		
Unmarried/not cohabitating	32/108	29·5 (19·0–39·9)	1·3 (0·7–2·5)			
Married/cohabitating	41/158	24·0 (17·0–31·0)	Ref			
**Ever widowed**				0·795		
No	49/178	25·5 (18·6–32·5)	0·9 (0·5–1·8)			
Yes	24/88	27·3 (15·6–39·0)	Ref			
**Education**				0·238		
No primary	6/39	16·3 (1·4–31·2)	Ref			
Complete primary	29/80	33·1 (21·6–44·5)	2·5 (0·7–8·9)			
Secondary +	38/147	24·3 (16·7–31·9)	1·6 (0·5–5·3)			
**Wealth index**				0·379		
Lowest	12/41	29·5 (11·5–47·4)	2·3 (0·6–8·3)			
Second	15/62	20·8 (9·5–32·1)	1·4 (0·4–4·7)			
Middle	15/53	29·0 (18·5–39·4)	2·2 (0·7–6·6)			
Fourth	23/69	34·5 (19·7–49·3)	2·9 (0·8–9·9)			
Highest	8/41	15·6 (2·7–28·4)	Ref			
**Missed doses**				0·086		
Yes	12/29	46·1 (25·7–66·5)	2·8 (1·1–7·2)		**2**·**6 (1**·**0–7**·**1)**	**0**·**049**
Unknown	19/67	24·3 (13·8–34·8)	1·0 (0·5–2·1)		0·9 (0·4–1·9)	0·694
No	42/170	23·4 (15·7–31·2)	Ref		Ref	
**Time in care**				0·825		
<24 months	12/45	22·6 (11·1–34·0)	0·8 (0·4–1·8)			
24+ months	41/151	26·9 (18·3–35·4)	1·0 (0·5–2·0)			
Unknown	20/70	26·6 (15·7–37·5)	Ref			
**TB co-infection**				0·561		
No	56/202	27·0 (19·7–34·3)	Ref			
Yes	17/64	23·2 (12·7–33·7)	0·8 (0·4–1·6)			
**Number of sex partners in past 12 months**				0·833		
None	21/83	24·3 (14·1–34·4)	Ref			
1	45/154	27·5 (19·7–35·2)	1·2 (0·6–2·2)			
2+	7/29	23·2 (6·1–40·3)	0·9 (0·3–2·8)			
**Condom use at last sex**				0·520		
Yes	27/105	23·5 (15·5–31·5)	Ref			
No	25/78	31·7 (19·5–44·0)	1·5 (0·7–3·2)			
Not sexually active	21/83	24·3 (14·1–34·4)	1·0 (0·6–2·0)			
**Consistent condom use with all partners in past 12 months**				0·889		
Yes	23/80	25·8 (16·7–35·0)	Ref			
No	29/103	27·6 (18·3–37·0)	1·1 (0·6–2·1)			
Not sexually active	21/83	24·3 (14·1–34·4)	0.9 (0·5–1·7)			

^1^Detectable viral load defined as HIV RNA concentration ≥550 copies/mL.

^2^Confidence Interval.

^3^Odds Ratio.

^4^Multivariate model adjusted for variables that were significant at <0·1 in the bivariate model: Age-group and missed doses.

In multivariate analysis for ART-experienced Kenyans, detectable viremia was independently associated with younger age and lack of adherence to ART. Young persons aged 15–29 years taking ART had three times the adjusted odds as persons aged 30–64 years to have detectable viremia (aOR 3·1, [95% CI:1·4–6·9]) (p <0·006) (Table 2). There were no other discernible differences across socio-economic or behavioural characteristics. However, sub-optimal adherence as measured by any missed ART doses in preceding 30 days tripled the adjusted odds of having a detectable VL (aOR2·6, [95% CI: 1·0–7·1]) (p = 0·049) compared to persons who reported they did not miss any doses in the preceding 30 days (Table 2).

### Geospatial analysis

**Fig 1:**
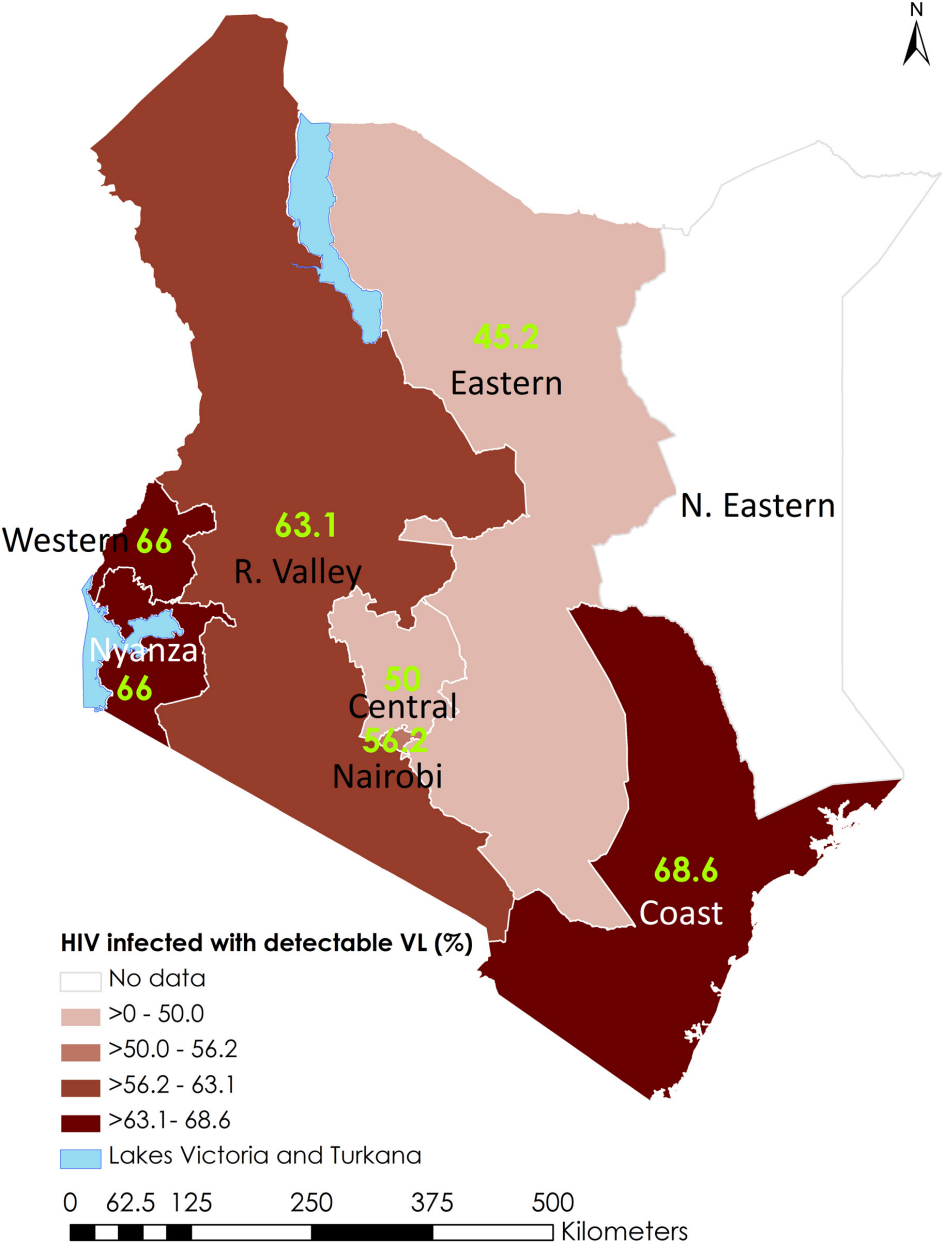
Distribution of detectable viral load^1^ by NASCOP region, Kenya AIDS Indicator Survey, 2012. NASCOP, National AIDS Control Programme. These regions correspond to former provinces of Kenya before the 2013 decentralization into counties. Detectable viral load defined as HIV RNA concentration ≥ 550 copies/mL.

Across all eight NASCOP regions, we estimated the proportion of HIV-infected persons who had detectable virus as a ratio of those who did not have detectable virus (Fig 1). Nationally there were 60% more HIV-infected people with detectable virus compared to those with PVL<550 copies/mL. We observed some heterogeneity of this ratio across the ten surveyed programmatic regions (varying from 0·8 in Eastern North to 2.7 in Rift Valley North) (Table 3). In Nyanza, the region with the highest burden of HIV in Kenya, there were half as many HIV-infected persons who were virologically suppressed compared to those who were not. Overall, Rift Valley, Western, Nyanza and Coast regions had ratios higher than the national average; whereas Nairobi, Central, and Eastern had ratios lower than the national average.

**Table 3:** Distribution of detectable and non-detectable viral load^1^ by NASCOP region, Kenya AIDS Indicator Survey, 2012.

NASCOP region	Overall	Undetectable VL <550 copies/uL		Detectable VL ≥550 copies/uL		
Region	No. HIV Infected	n	wt^2^ % (95% CI^3^)	n	wt^2^ % (95% CI^3^)	Ratio
**National**	617	235	38.8 (33.9–43.6)	382	61.2 (56.4–66.1)	1.6
**Nairobi**	63	26	43.8 (29.9–57.8)	37	56.2 (42.2–70.1)	1.28
**Central**	57	31	50.0 (34.7–65.3)	26	50.0 (34.7–65.3)	1.0
**Coast**	63	20	31.4 (16.2–46.6)	43	68.6 (53.4–83.8)	2.2
**Eastern—North**	28	13	50.4 (27.3–73.6)	15	49.6 (26.4–72.7)	0.98
**Eastern—South**	45	25	55.0 (38.8–71.2)	20	45.0 (28.8–61.2)	0.82
**Nyanza**	227	76	34.0 (27.3–40.6)	151	66.0 (59.4–72.7)	1.9
**Rift Valley—North**	36	10	27.9 (12.7–43.1)	26	72.1 (56.9–87.3)	2.7
**Rift Valley—South**	41	15	42.6 (19.2–66.1)	26	57.4 (33.9–80.8)	1.3
**Western**	57	19	34.0 (20.0–48.0)	38	66.0 (52.0–80.2)	1.9

NASCOP, National AIDS Control Programme. These regions correspond to former provinces of Kenya before the 2013 decentralization into counties.

^1.^ Detectable viral load defined as HIV RNA concentration ≥ 550 copies/mL. Undetectable viral load defined as HIV RNA concentration < 550 copies/mL.

^2.^ Weighted.

^3.^ Confidence Interval.

Among those with detectable viremia, 37·4% knew their HIV-positive status, half (49·9%) of whom had not initiated ART. Although the sample size was small (N = 31), 59·3% (95% CI 38-4-80.1) of ART-naïve participants with detectable viral load had CD4+ T-cell count measurements >500 cells/μL indicating they were ineligible for ART under the current Kenya guidelines. Even then, among ART-experienced persons, 62·2% had CD4+ T-cell counts = <350 cells/ μL.

## Discussion

To the best of our knowledge, this is the first national survey to report population-level virological suppression in Kenya. We found high levels of viremia among HIV-infected Kenyans who were not currently taking ART. Furthermore, we demonstrated geospatial differences in the distribution of viremia. Our report adds to the limited data on population viral load [15, 16].

Our data compare well with previous reports of virologic suppression. In our study 38·8% of all HIV-infected persons and 73·9% of ART-experienced persons had suppressed viremia. In Swaziland, viral suppression was 35% and 85% among all HIV-infected persons and among those on ART respectively, similar to results from a sub-national household survey in Kenya that reported that 39.7% of HIV-infected adults had less than 1000 copies/ml, with 83.6% of those on ART being virologically suppressed [15, 16]. In the US, it is estimated that between 19%-29% of people living with HIV/AIDS are optimally suppressed [17, 18]. The differences in these estimates result from disparate methodologies for measuring VL including VL cut-offs and underlying differences in ART access and knowledge of HIV status. In the US, it is estimated that in 2010, 16% of persons with HIV infection were unaware of their HIV status, compared to 42% of those in Kenya in 2012. Still, our data validate findings that treatment outcomes in low-income countries including adherence to ART and virologic suppression are commensurate to those in high-income countries [19, 20]. However, substantial efforts are needed to reach the Joint United Nations Programme on HIV and AIDS (UNAIDS) 90-90-90 treatment targets and achieve 90% viral suppression among people living with HIV on ART by the end of 2020 [21]. In this study we demonstrate that the majority of ART-naïve patients who are aware of the HIV-positive status have CD4+ T-cell counts >500 cells/ μLand are therefore not eligible for ART as per current Kenya national guidelines. To meet the UNAIDS goals, expansion of eligibility criteria for ART would potentially achieve wider population-level viral suppression.

In our study, we confirm that lack of knowledge of HIV-positive status, not being on ART and sub-optimal adherence is an important determinant of detectable viremia, similar to findings reported in other settings [11, 15]. Among persons on ART, virological failure has been associated with concurrent TB infection [22], and recent incarceration [23]. In our study we did not detect any difference in viral suppression among TB patients on ART. This could be due to the fact that TB diagnosis is a vital criterion for initiation of ART in Kenya.

It is also evident that lack of knowledge of status is a strong barrier for accessing ART [24]. Without HIV testing, timely initiation of ART and retention in care, the goal of elimination of sexual transmission as a global public health imperative will be difficult to realize [25, 26]. Additionally to achieve population-level viral suppression, cheaper and more potent ART, increased laboratory capacity, and significant investments in health systems are required. In particular, VL measurements are cost-effective for routine use in clinical settings to monitor ART success [27, 28], and are increasingly proposed as a standard of care for initiation of ART [29] and as a HIV prevention outcome [25]. Widespread availability of high quality point-of-care VL testing is therefore warranted.

In our study we demonstrated that there is heterogeneity in viremia across geographical regions. As HIV evolves from a generalized to a concentrated epidemic, a geospatial approach to surveillance and implementation of HIV programs provides important insights into sub-national variations of the HIV epidemic. This would enable identification of drivers of the epidemic to inform geographic prioritization and resource allocation [30]. Geospatial analysis is consistent with UNAIDS goal of maximizing the efficiency and impact of national HIV prevention programs by focusing on locations and populations with the highest HIV burden especially in resource-limited settings [31].

Our paper has several limitations. We used a PVL cut-off of 550 copies/mL as the limit of detection yet the World Health Organization defines suppression as RNA concentrations <1000 copies/mL for routine monitoring; other publications have used <400 copies/mL or even lower thresholds as the technology to detect low levels of viremia has improved [32]. These differences limit our ability to compare rates of virologic suppression across different settings. It would be helpful to standardize VL metrics and VL assays for global comparability of estimates. Although we reported median and mean VL, the median VL has minimal utility in jurisdictions that have successfully scaled-up ART and the mean VL is prone to influence of outlying measurements. Both of these are not appropriate to quantify population-level infectivity. Due to a small sample size, we did not have sufficient power to detect differences in prevalence of viremia at the county level where management of health programmes has devolved in Kenya. Because our study relied on participant self-report, responses to sensitive questions such as ART use and testing history may have been biased towards more socially desirable answers, potentially leading to an under-representation of the true coverage of ART use in the population and overestimation of suppression among untreated persons. We believe that the addition of biological confirmation of ART to define “current ART use” in our analysis helped to reduce this potential bias. Given the level of VL suppression found among persons who were not on ART, our data support that VL suppression may not necessarily equal to ART-related suppression. Only half of CD4+ T-cell count tests were available due to hemolysis of specimens and hampered broader interpretation of viral suppression among those aware of their HIV-infection.

Despite these limitations, the strength of our analysis is the nationally representative sample that was used to expand our knowledge of population level coverage of HIV VL in Kenya. Furthermore this is a benchmark for future trends in VL measurements among ART-naïve and ART-experienced persons and will enable time-series analysis of VL and supplement HIV incidence estimates in Kenya. We recommend that VL monitoring be an essential component of national surveillance strategies, integrated in national monitoring and evaluation and health information systems to monitor trends of infectivity at the population level.
